# The folding interactome of GPCRs

**DOI:** 10.1186/1471-2210-11-S2-A41

**Published:** 2011-09-05

**Authors:** Christian Bergmayr, Christian Nanoff, Oliver Kudlacek, Michael Freissmuth, Christian W Gruber

**Affiliations:** 1 Institute of Pharmacology, Center of Physiology and Pharmacology, Medical University of Vienna, 1090 Vienna, Austria

## Background

The A_2A_ adenosine receptor is a prototypical G protein-coupled receptor. It is expressed in a wide variety of cells including as different types as neurons, platelets, cells of the immune system and muscle. The A_2A_ receptor has an unusually long C-terminus (of >120 residues), which for the most part is dispensable for coupling to G_s_. This C-terminus turned out to be the docking site for other proteins. Using a yeast-2-hybrid screen we have previously identified proteins interacting with the C-terminus including ARNO/cytohesin2, SAP102 and USP4.

## Methods

To verify these interactions *in vivo* and to identify new interacting proteins of the A_2A_ adenosine receptor we chose a two-step proteomics approach: we first expressed tagged receptors in HEK293 fibroblasts using various TAP (tandem affinity purification)-tag variants; the differently tagged receptors were analyzed for expression, localization and their pharmacological properties (ligand binding and cAMP accumulation) to identify tags suitable to further analyze the receptor’s interactome. These tagged receptors were then used to optimize the purification and to make the first initial screens using 2D-nano-LC-MS/MS approach. To prove the interaction of the A_2A_ receptor with promising targets found in our screens, biochemical approaches, e.g. co-immunoprecipitation and whole-cell binding, were performed.

## Results and conclusions

We could identify two tags suitable for further analysis of the A_2A_ adenosine receptor interactome. Pharmacological properties of the tagged receptors were comparable to the native receptor. However, the tags seemed to retain the receptor to a large extent in the endoplasmic reticulum (ER) and hence we used this system to study the ER/folding interactome of the receptor. LC-MS/MS analysis of the purified ER-trapped version of the receptor revealed proteins putatively involved in the folding of the receptor, such as chaperones. We are currently generating a transgenic mouse-model expressing the TAP-tagged version of the A_2A_ adenosine receptor under the control of its endogenous promotors (homologous knock-in). This will allow us to examine tissue- and development-specific interaction partners of the A_2A_ adenosine receptor utilizing the optimized proteomics approach.

